# Sexual Dimorphism Floral MicroRNA Profiling and Target Gene Expression in Andromonoecious Poplar (*Populus tomentosa*)

**DOI:** 10.1371/journal.pone.0062681

**Published:** 2013-05-07

**Authors:** Yuepeng Song, Kaifeng Ma, Dong Ci, Zhiyi Zhang, Deqiang Zhang

**Affiliations:** 1 National Engineering Laboratory for Tree Breeding, College of Biological Sciences and Technology, Beijing Forestry University, Beijing P. R. China; 2 Key Laboratory of Genetics and Breeding in Forest Trees and Ornamental Plants, College of Biological Sciences and Technology, Beijing Forestry University, Beijing, P. R. China; Pennsylvania State University, United States of America

## Abstract

Although the molecular basis of poplar sex-specific flower development remains largely unknown, increasing evidence indicates an essential role for microRNAs (miRNAs). The specific miRNA types and precise miRNA expression patterns in dioecious plant flower development remain unclear. Here, we used andromonoecious poplar, an exceptional model system, to eliminate the confounding effects of genetic background of dioecious plants. This system, combined with high-throughput sequencing and computational analysis, allowed us to characterize sex-specific miRNAomes from female and male flowers. Comparative miRNAome analysis combined with quantitative real-time PCR revealed the expression patterns of 27 miRNAs in poplar flower and showed that the targets of these miRNAs are involved in flower organogenesis, Ca^2+^ transport, phytohormone synthesis and metabolism, and DNA methylation. This paper describes a complex regulatory network consisting of these miRNAs expressed in sex-specific flower development in a dioecious plant. The conserved and novel miRNA locations were annotated in the *Populus trichocarpa* genome. Among these, miRNA Pto-F70 and 4 targets are located in the sex-determination regions of chromosome XIX. Furthermore, two novel miRNAs, Pto-F47 and Pto-F68, were shown for the first time to be regulatory factors in phytohormone interactions. To our knowledge, this report is the first systematic investigation of sex-specific flower-related miRNAs and their targets in poplar, and it deepens our understanding of the important regulatory functions of miRNAs in female and male flower development in this dioecious plant.

## Introduction

The genus *Populus* encompasses approximately 100 species divided into 5 sections and many of these species are important components of terrestrial ecosystems or important cultivated trees for wood, pulp, paper, and possibly biofuels uses. As dioecious trees, the genus *Populus* provides opportunities to study perennial woody plant biology and plant sexual dimorphism [1]. Moreover, the high-quality, annotated genome sequence of *P. trichocarpa* provides a key genomics tool, enabling genome-wide regulatory analyses [1]. Sexual dimorphism is a widely studied phenomenon in dioecious plants and likely results from different modes of selection operating in males and females [2]. As dioecious trees, poplar floral development is sexual dimorphism. The poplar flower is composed of two whorls, including a reduced perianth cup surrounding either the stamens or carpels, which begin to develop in spring (early May in Shandong province) of the year before the spring in which they will open [3], [4]. Until late May or early June, structural development of the male inflorescence is virtually indistinguishable from development of the female inflorescence. After that, the flower morphology, bract morphology, and cell division patterns differ markedly between the sexes [5]. Pioneering experiments found that a series of genes involved in flowering pathways are differently expressed between female and male flowers [6], [7]. Although the molecular basis of poplar sex-specific flower development remains largely mysterious, increasing evidence suggests that miRNAs may play essential roles in this process.

In plants, there are two classes of endogenous small RNAs: small interfering RNAs (siRNAs) and microRNAs (miRNAs). Both classes, ranging from 20–24 nt, are short non-coding RNA molecules that negatively regulate gene expression at the transcriptional and/or post-transcriptional levels [8]. siRNAs are generated from double- stranded RNA. By contrast, miRNAs are transcribed from a long precursor molecule that folds back on itself to form a hairpin. Dicer-Like1 (DCL1) protein cleaves this precursor molecule, resulting in a miRNA:miRNA* complex, which separates into miRNA and miRNA* after transport to the cytoplasm [9]. In the cytoplasm, the miRNA is bound by ARGONAUTE proteins to form part of the RNA-induced silencing complex (RISC), which interacts with target mRNAs to cleave the RNA of target genes at the paired region [10]. Since the mature miRNA and its complementary target sequence have almost perfect complementarity, identifying a miRNA usually leads to the prediction and/or identification of its target. miRNAs have been shown to target genes during plant organ development, stress tolerance, phytohormone signaling, growth phase change, and disease resistance [11], [12].

miRNAs have been identified as regulators of flower development and other pathways. For example, in *Arabidopsis*, the miR164 family targets a subset of NAC transcription factors including *CUP-SHAPED COTYLEDON1* (*CUC1*) and *CUC2*, which mediate organ boundary formation. Among these members, miRNA164c affects petal number in early flowers [13]. miRNA172 regulates *APETALA2* (*AP2*) and several of its homologs that share two tandem AP2 DNA-binding domains and play an important role in regulating flower development [14]–[16]. In maize, *TASSELSEED 4* (*TS4*) and the *AP2* homolog *indeterminate spikelet 1* are confirmed miRNA172 targets. In *ts4* mutants, female organs develop in the male inflorescence. Moreover, branching is increased in *ts4* mutants indicating a link between sex determination and meristem fate [17]. In tree species, the unknown functions of the majority of genes are still the greatest gap. miRNA studies provided a best chance for directing to identify functions for genes [18]. Small RNA libraries prepared from poplar leaves and vegetative buds have been analyzed by high-throughput sequencing, finding 48 *Populus*-specific miRNA families [19]. However, sex-specific miRNA differences between female and male flower organs remain unclear, partly because of the effects of different genetic backgrounds of dioecious plants. For this work, we first used andromonoecious poplar, an exceptional model system that has both male and hermaphrodite flowers on a single plant, to eliminate confounding effects of genetic background of dioecious plants (Figure S1). In this study, our aim is to screen sex-specific potential functional miRNAs in poplar floral tissue, investigate their defined expression patterns and demonstrate a possible functional network of sex-specific miRNAs in poplar flower organs.

## Results

### Small RNA Sequence Analyses

**Figure 1:**
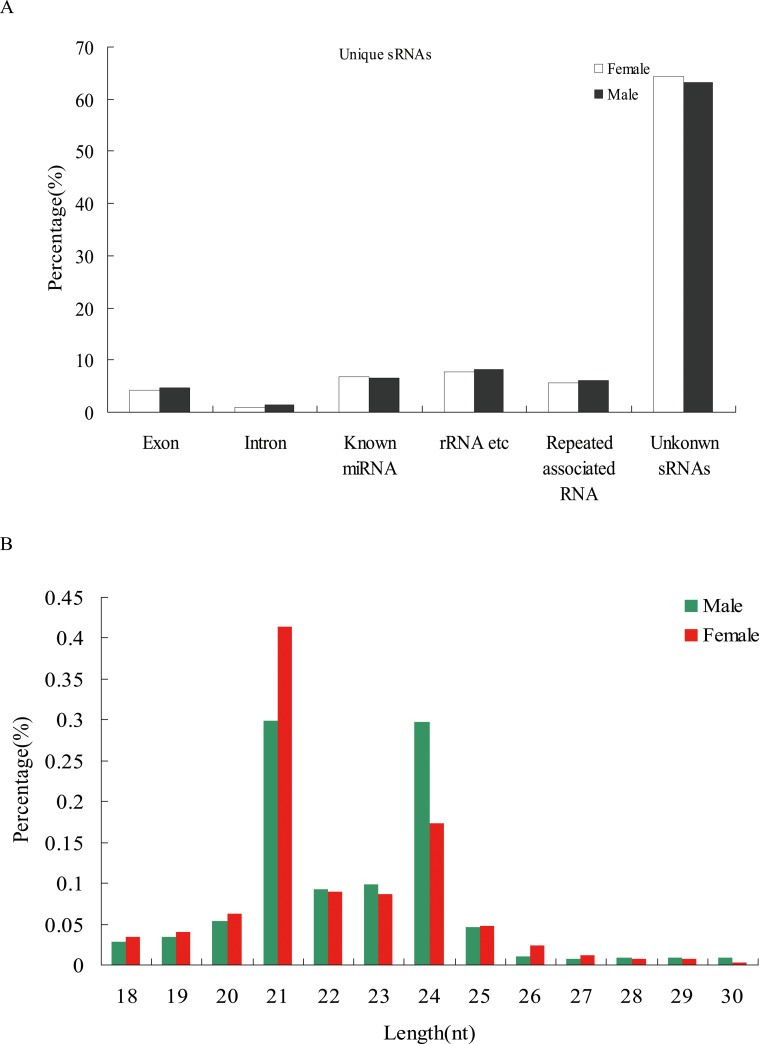
Small RNAs from female and male flower libraries in andromonoecious poplar. (A) Distribution of unique sRNA annotation categories. (B) Size distribution of small RNA reads.

Two small RNA (sRNA) libraries were constructed from female (F) and male (M) flowers of andromonoecious *P*. *tomentosa*. A total of 58,517,150 (F) and 47,918,351 (M) raw reads were generated from these libraries, respectively, using the Solexa sequencing technology (Table 1). After removing contaminant reads, clean reads were obtained and screened against rRNA, tRNA, snRNA, snoRNA, and mRNA in the Rfam 9.1 and NCBI GenBank databases, resulting in 50,782,479 (F) and 42,837,470 (M) reads remaining for further analyses. Conserved miRNAs were identified by alignment to data in miRBase 19.0 with ±2 nt mismatch; a total of 4,789,550 (F) and 4,306,015 (M) reads that could form characteristic hairpin structures were identified. 4,459,932 (F) and 3,810,615 (M) unannotated unique sRNA sequences, which were further analyzed to predict novel miRNAs. The sequenced sRNAs were then mapped to the *Populus* genome, miRBase19.0, the NCBI GenBank database, and the Rfam database, and classified into six categories: rRNA, known miRNA (miRNAs in miRBase 19.0), exon, intron, repeat associated RNA, and unknown sRNA. More than 60% of the sequences were unknown sRNAs (Figure 1A). The majority of total sRNA reads were either 21 or 24 nt for both libraries (Figure 1B). In female flower libraries, the 21-nt sRNAs were the most abundant, making up 41.4% of total sequence reads. The 24-nt sRNAs were the second most abundant class at 17.3% of total sequence reads. In male flower libraries, the 21 and 24 nt sRNAs were not significantly different, making up 29.9% and 29.7%, respectively.

**Table 1:** Summary of small RNA sequences from *P*. *tomentosa* female and male flower.

	Female library (F)	Male library (M)
Raw reads	58,517,150	47,918,351
Clean reads	50,782,479	42,837,470
Annotated sRNAs	16,625,919	16,030,471
Un-annotated sRNAs	34,156,560	26,806,999
Small RNAs	4,789,550	4,306,015
Annotated sRNAs	329,618	495,400
Un-annotated sRNAs	4,459,932	3,810,615

### Conserved miRNAs in Andromonoecious *P. tomentosa*

**Figure 2:**
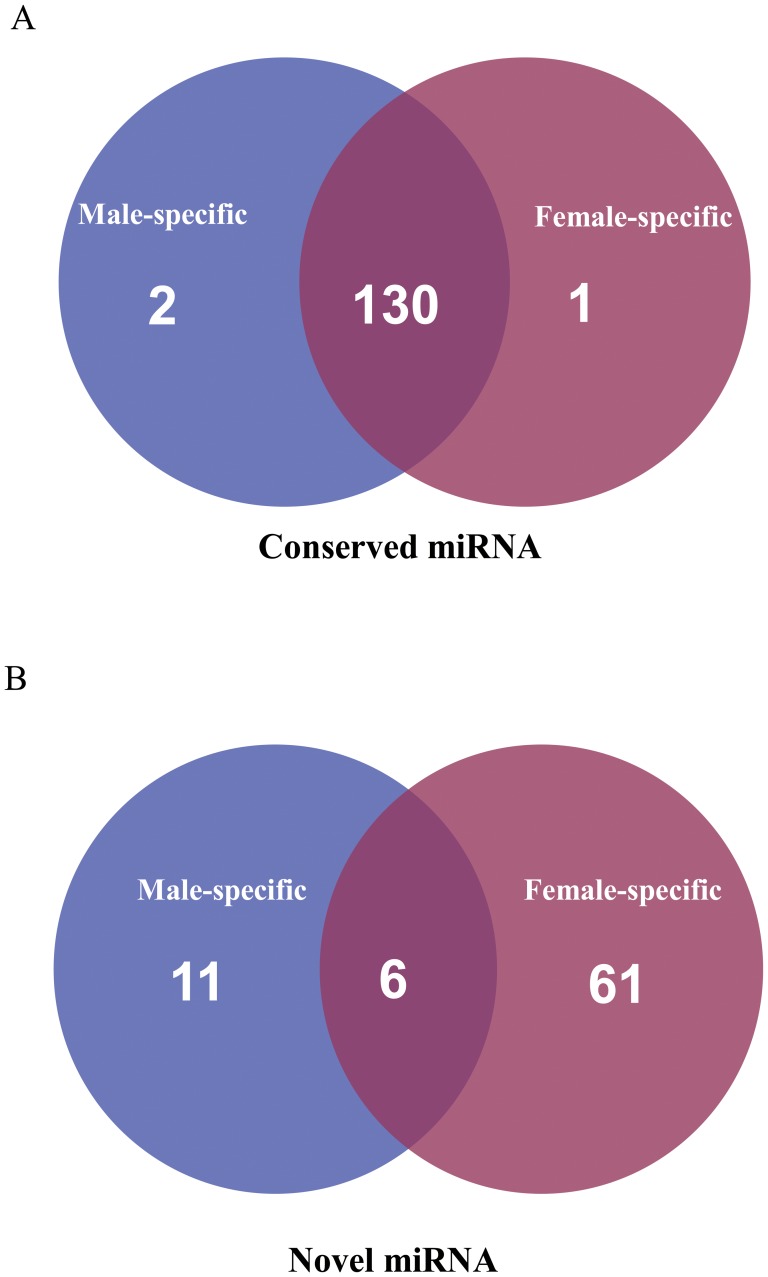
Summary of common and specific sequences between female and male libraries. (A) Conserved sRNAs and (B) Novel sRNAs.

To date, 46 conserved and non-conserved miRNA families have been discovered in the *Populus* genome [20]. In the two libraries sequenced in our study, 134 unique miRNA sequences were identified belonging to 38 conserved miRNA families (Table 2). The expression levels of a few miRNA families, such as miR166 and miR472, were extraordinarily high in both libraries (Table 2). miR166 was the most abundant, with 2,157,665 (F) and 142, 916 (M) reads accounting for 62.5% and 47.1% of all conserved miRNA reads, respectively. Several miRNA families, such as miR156, miR159, miR169, miR319, miR396, and miR1447, had moderate expression levels (Table 2). By contrast, some miRNA families showed very low levels of expression, with fewer than 200 reads. Different members in the same miRNA family displayed drastically different expression levels. For example, miR166 members varied in abundance from 695 to 2,157,665 reads. Also, 38 unique miRNA sequences belonging to 38 conserved miRNA families were produced from 134 loci (Table S1). Of these miRNA sequences, 11 were encoded by a single locus in the *Populus* genome, whereas the other 27 sequences had multiple loci. Most of these had 2–6 loci in the genome, and only a few had more than eight loci. For example, Pto-miR166a–l and Pto-miR169a–m had 12 and 13 loci, respectively. Thus, the size of miRNA families varies in andromonoecious *P. tomentosa*. Among these conserved miRNAs, miR6459 was only expressed in the female library. By contrast, two miRNAs, miRNA397 and miRNA6462, were detected as having male-specific expression (Figure 2).

**Table 2:** Summary of conserved miRNAs found in andromonoecious *P. tomentosa*.

miRNAfamily	Members	Reads		Ratios (female/male)
		Female	Male	
miR156	6	3629	8330	0.44
miR159	3	808474	20798	38.87
miR160	4	2695	523	5.15
miR162	2	5094	342	14.89
miR166	12	2157665	142916	15.10
miR167	8	1995	15568	0.13
miR168	2	38333	5129	7.47
miR169	13	41555	3514	11.83
miR171	5	820	298	2.75
miR172	2	58	105	0.55
miR319	6	115137	2239	51.42
miR393	5	2498	1911	1.31
miR394	2	4814	1143	4.21
miR396	9	30598	30786	0.99
miR397	1	0	8	0.00
miR398	3	1783	752	2.37
miR399	10	11180	116	96.38
miR403	3	19815	5087	3.90
miR408	1	157	161	0.98
miR472	2	126848	48300	2.63
miR475	7	1524	394	3.87
miR476	3	1101	637	1.73
miR482	2	1973	312	6.32
miR530	1	337	11	30.64
miR1444	1	29	36	0.81
miR1446	4	22	51	0.43
miR1447	1	22381	2700	8.29
miR1448	1	16637	4935	3.37
miR1450	1	16340	826	19.78
miR6421	1	641	711	0.90
miR6425	4	219	245	0.89
miR6427	1	345	112	3.08
miR6433	2	1026	374	2.74
miR6445	2	9334	3382	2.76
miR6447	1	2	10	0.20
miR6459	1	603	0	0.00
miR6462	1	0	5	0.00
miR6478	1	1846	1028	1.80
Total	134	3447508	303795	11.35

### Identification of Novel miRNAs in Andromonoecious *P. tomentosa*

**Figure 3:**
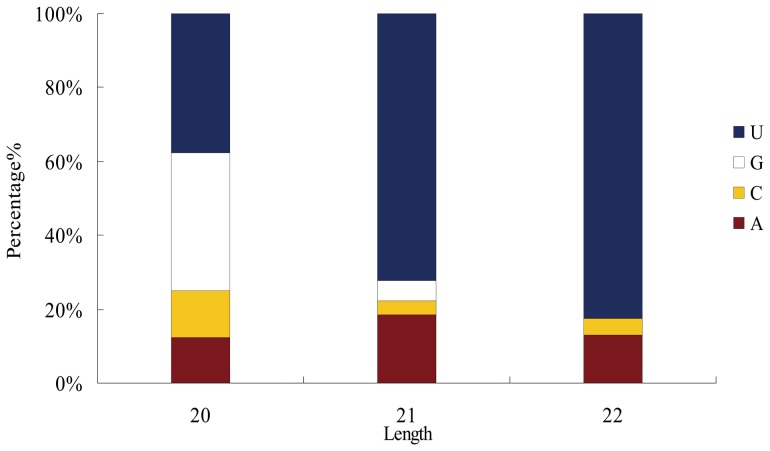
First nucleotide bias of novel candidate miRNAs in andromonoecious poplar female and male flower libraries.

The characteristic hairpin structure of miRNA precursors was used to predict novel miRNAs. In total, 78 unique sRNA sequences were identified including 8 sRNAs with complementary miRNA*s (Table 3, 4 and 5). The majority of these novel miRNA candidates had lengths of 21 and 22 nt, and started with a 5′ U (Figure 3). The 78 sRNA sequences were transcribed from 85 loci; most were only produced from one locus, but Pto-F2, Pto-F13, Pto-F31 and Pto-F31 were each generated from 3 loci. The length of the predicted novel miRNA precursors ranged from 76 to 303 nt, with an average of 140 nt. The average minimum free energy (MFE) value was −55.58 kcal/mol, with a range of −21.2 to −177.4 kcal/mol. The structures of the 78 novel miRNA precursors are shown in Figure S2. Most of them showed differential expression in the two libraries. For instance, the mature miRNA counts ranged from 63 to 8,480,860. Among those 78 miRNAs, only 6 sRNAs were found both in female and male libraries. 61 female-specific sRNAs and 11 male-specific sRNAs were detected, suggesting that more kinds of sRNA are involved in female floral development than in male development. To investigate whether these novel miRNA sequences were conserved across plant species, we used them as query sequences to perform BLASTN searches against the *Arabidopsis thaliana*, *Brassica rapa*, *Citrus sinensis*, *Medicago truncatula*, *Oryza sativa*, *Ricinus communis*, *Prunus persica*, *Vitis vinifera* and *Zea mays* genome databases in Phytozome v7.0. A large proportion of the novel sRNAs identified in andromonoecious *P. tomentosa* did not have perfect matches in different genome databases. Only 6 male-specific sRNA and 5 female-specific sRNAs were detected in other species (Table S2).

**Table 3:** Summary of common novel miRNAs in female and male flower libraries.

Name	Sequence (5′-3′)	LP	MFE	miRNA location	miRNA reads		Ratio(Female/Male)	miR*reads
					Female	Male		
Pto-F1	ATAGGTTTCCGATCATTCCTCC	169	−81.7	chr13∶103779–103800	8990	391	22.99	NO
Pto-F2a	TGAAATGCCATGAACAACGATC	125	−53.4	chr13∶11993451–11993472	433	8111	0.05	NO
Pto-F2b	TGAAATGCCATGAACAACGATC	115	−47	chr16∶4142967–4142987	377	942	0.40	NO
Pto-F3	ATGTAATCAAGGCTGCTAGTC	141	−72.3	chr17∶8682859–8682879	71	434	0.16	Yes
Pto-F4	TCTAAAGATCCGGCGGTCGGC	124	−44	chr18∶2804698–2804718	63	90	0.70	NO
Pto-F5	TATGTTTGACAGAAACCCCCT	171	−89.2	chr18∶8350113–8350132	729	1991	0.37	NO
Pto-F6	TGTGGTAGATATGTGAGGATT	148	−65.2	chr19∶10983127–10983147	472	233	2.03	NO

**Table 4:** Summary of male-specific novel miRNAs.

Name	Sequence (5-3)	LP	MFE	miRNA location	miRNA reads	miR*reads
Pto-F7	CTCAAACTTTTCTCTTACTTC	283	−168.1	chr2∶1282971–1282991	147	NO
Pto-F8	TACCGTTAGAAAGATCTCAAC	179	−62	chr2∶8266846–8266866	126	NO
Pto-F9	ATAGGTTTCCGATCATTCCTCC	199	−85.04	chr5∶119707–119728	819	YES
Pto-F10	TTTTGCACGTAGAATTCAGGA	139	−38	chr5∶18398105–18398125	147	NO
Pto-F11	ACGAGCCATCATAACTGTAGG	125	−81.92	chr6∶6767480–6767500	714	YES
Pto-F12	TAGTTCGAGGGACCAAAATCA	219	−111.4	chr6∶8063061–8063081	357	YES
Pto-F13	ATCTGCTTTCTGCGACTCCTC	183	−68.8	chr8∶8028138–8028158	126	NO
Pto-F14	GCATTTGGACGTCGGGGAACT	90	−23.7	chr9∶7157296–7157316	357	NO
Pto-F15a	AGGATTGGAGGGAATTAAACA	135	−40.55	chr10∶20020250–20020270	105	NO
Pto-F15b	AGGATTGGAGGGAATTAAACA	129	−41.7	chr12∶11845073–11845093	105	NO
Pto-F16	TAATTCCATGACTGTGTACAG	156	−37.82	chr15∶10265155–10265175	189	NO
Pto-F17a	TTTAATTTCCTCCAATATCTTA	133	−45.54	chr18∶14578403–14578424	210	NO
Pto-F17b	TTTAATTTCCTCCAATATCTTA	136	−45.54	scaffold_269∶8974–8995	210	NO
Pto-F17c	TTTAGTTTCCTCCAATATCTTA	136	−41.2	scaffold_269∶9454–9475	189	NO

**Table 5:** Summary of female-specific novel miRNAs.

Name	Sequence (5-3)	LP	MFE	miRNA location	miRNA reads		miR*reads	
Pto-F18	TTGGTTAAACTCCCATCAGGA	133	−65.6	chr1∶7117690–7117710	126		NO	
Pto-F19	CTCAGATTAGCCAGGTGCCT	117	−44.3	chr1∶10983482–10983501	105		NO	
Pto-F20	TTATTAAACCCGGACCGGCCT	122	−54.62	chr1∶18312617–18312637	126		NO	
Pto-F21	TACCCGGTTACTGTGCATGTGC	76	−33.3	chr1∶38996098–38996119	126		NO	
Pto-F22	TATCCTTGGATCTGACGACCA	220	−123.4	chr1∶39819960–39819980	294		NO	
Pto-F23	TTTTCGATCCAAGCTTCGGGT	80	−27.1	chr1∶42987729–42987749	105		NO	
Pto-F24	TTACTTCCTTTTGTCCCTCTC	303	−177.4	chr2∶1282957–1282977	357		NO	
Pto-F25	TATAGATTGCAGAGGGAACC	203	−71.04	chr2∶12233873–12233892	126		NO	
Pto-F26	TTATTAAACCCGGACCGGCCT	78	−42.8	chr2∶19952785–19952805	126		NO	
Pto-F27	TCCGAGCTCTAATTATGTGGG	182	−92	chr2∶21648214–21648234	798		NO	
Pto-F28	TTGGGCTGGCAGTTGTGATGAC	161	−45.87	chr3∶19076220–19076241	231		NO	
Pto-F29	TTCCGGTTTTTGGGACTCCGAT	103	−35.1	chr4∶20319258–20319279	105		NO	
Pto-F30	TTCTTTCCTATCAAACTTCAAC	191	−92.4	chr4∶22210258–22210279	105		NO	
Pto-F31	TGAAGATAAGAGCTTGTTTGG	169	−75.86	chr5∶6548077–6548097	651		NO	
Pto-F32	TTTGCACGTAGAATTCAGGATT	139	−38	chr5∶18398103–18398124	588		NO	
Pto-F33	ATGGATTGATGAAGATGAGGA	92	−26.3	chr5∶21111319–21111339	105		NO	
Pto-F34	TTATTTCCTCAAACTTTCCTC	256	−159.8	chr5∶24262419–24262439	273		NO	
Pto-F35	TAGAACCTGACCAGTTGAGCT	116	−30	chr6∶2313141–2313161	105		NO	
Pto-F36	TGGCCCATGATCTTCATTGTG	115	−78.02	chr6∶6767559–6767579	357		YES	
Pto-F37	TTTTTGTTGTTTAAGAACCCT	301	−128.6	chr6∶21307737–21307757	315		NO	
Pto-F38	TTGGGCTGGCAGTTGTGATGAC	161	−46.2	chr7∶301518–301539	231		NO	
Pto-F39	TGTGGGACTCGAATTAGTGAT	96	−26.43	chr7∶13307862–13307882	147		NO	
Pto-F40a	GAGGAGGGAGAGAGATCTGT	136	−38.3	chr8∶5498368–5498387	126		NO	
Pto-F40b	GAGGAGGGAGAGAGATCTGT	114	−34.3	chr8∶5522852–5522871	126		NO	
Pto-F41	ATCCAACGGTTAGATCTCCCT	296	−162.7	chr8∶8443078–8443098	105		NO	
Pto-F42	CTTGAAACAATGTTTGGTGCAG	121	−31.9	chr8∶10699331–10699352	105		NO	
Pto-F43	TAGAGTATAAGAGGATCGACT	124	−32.4	chr8∶15869891–15869911	147		NO	
Pto-F44	ACTTTGACCTGAACTTGCCC	79	−40	chr9∶8255922–8255941	105		NO	
Pto-F45	TGTCCTGACTCGAACTCGAGA	82	−21.2	chr10∶7102937–7102957	189		NO	
Pto-F46	TGAAGATAAGAGCTTGTTTGG	169	−77.06	chr10∶7227906–7227926	651		NO	
Pto-F47	GGGACTGCTGTAGATGCTTGG	90	−34.7	chr10∶13462743–13462763	105		NO	
Pto-F48	TTGATGACTGATCTTGAGCAT	99	−62.8	chr10∶16376128–16376148	189		YES	
Pto-F49	TTGTAAGAGCCTGAGACAGCC	143	−51.8	chr10∶18334850–18334870	714	NO		
Pto-F50	TTTAATTTCCTCCAATATCTCA	169	−45.91	chr10∶20020296–20020317	399	NO		
Pto-F51	TTGGAATCCTCTCTGATAATGC	105	−30.5	chr10∶20322598–20322619	105	NO		
Pto-F52	TTTAGGACCCGTGGGATTAGTC	102	−27	chr11∶8039096–8039117	189	NO		
Pto-F53	TCAATGGGTAAAAATTCTGACC	96	−24.4	chr12∶3834076–3834097	126	NO		
Pto-F54	TTGGAGCTCGAGACTTGGCAC	90	−25.74	chr13∶1347925–1347945	105	NO		
Pto-F55	ATCACTGTGTCTATTAGGATGG	82	−28.1	chr13∶6333415–6333436	105	NO		
Pto-F56	TGCTAGGACCAAGTTTTCTGG	103	−53.34	chr13∶9970875–9970895	609	YES		
Pto-F57	TTCAGGACCTGTAGAATTAGTC	92	−24.2	chr13∶12124499–12124520	105	NO		
Pto-F58a	TGAATGGCTGTTGAACTTGGC	156	−49.5	chr14∶293794–293814	168	NO		
Pto-F58b	TGAATGGCTGTTGAACTTGGC	158	−45.51	chr14∶726835–726855	168	NO		
Pto-F58c	TGAATGGCTGTTGAACTTGGC	158	−52.6	chr14∶1080042–1080062	168	NO		
Pto-F59	TGATGGGTCTCATTTAGTAGA	215	−86.36	chr14∶5877228–5877248	441	NO		
Pto-F60	AACCGAACCGAACTGAACCGA	98	−33.4	chr14∶16267732–16267752	105	NO		
Pto-F61	TTATAGTTTTGAAATCCGGCC	95	−47.2	chr15∶5715046–5715066	147	NO		
Pto-F62	AACCCAAGAACTCAACTCTGT	171	−68.37	chr15∶6997081–6997101	126	NO		
Pto-F63	GTACGATCCTATAGAACAGAT	116	−34.5	chr15∶15015652–15015672	189	YES		
Pto-F64	CAACACCAGACCCAAGAGCTT	117	−44.51	chr16∶5544896–5544916	210	NO		
Pto-F65	TTAGGGTTTAGGGTTTAGAA	157	−41.6	chr17∶210550–210569	126	NO		
Pto-F66	TCGAGATTGTACTGTTCATA	77	−23.6	chr17∶4120360–4120379	105	NO		
Pto-F67	TGGCAAATGATCTGCATCTGC	90	−24.52	chr17∶13666346–13666366	105	NO		
Pto-F68	GTGGGTGGGTCTGGGTGGCA	118	−47.7	chr18∶10507789–10507808	147	NO		
Pto-F69	TTTGCTCTTCGTTTTCTCATG	170	−77.8	chr18∶14336738–14336758	294	NO		
Pto-F70	TTTTATTCCTGACTCTGGCATC	166	−43.31	chr19∶907650–907671	126	NO		
Pto-F71	TTGGGAATTATTACAATGGCA	106	−31.8	chr19∶1460855–1460875	147	NO		
Pto-F72	TTATTAAACCCGGACCGGCCT	87	−39.6	chr19∶2013039–2013059	126	NO		
Pto-F73	TGTTTTCCGGAAAGTAGTTTC	99	−27.1	chr19∶8890465–8890485	378	NO		
Pto-F74	TAGGGCAGAGAATTGAATGACT	119	−45.4	chr19∶10646670–10646691	252	NO		
Pto-F75	TCGAGATTTCTTTGTTGGAGCT	125	−42.72	scaffold_1233∶2309–2330	126	NO		
Pto-F76	TCGGCGTTGATGTAGAATGGC	153	−53.1	scaffold_401∶10122–10142	126	NO		
Pto-F77	TTTTGAGACAATGCTGAAAAT	99	−28.4	scaffold_65∶42511–42531	1953	NO		
Pto-F78	ACATGAAACGGCGTCGTTTTG	91	−26	scaffold_76∶39681–39701	126	NO		

### Novel miRNA and Targets were Located in Gender Determination Regions

**Figure 4:**
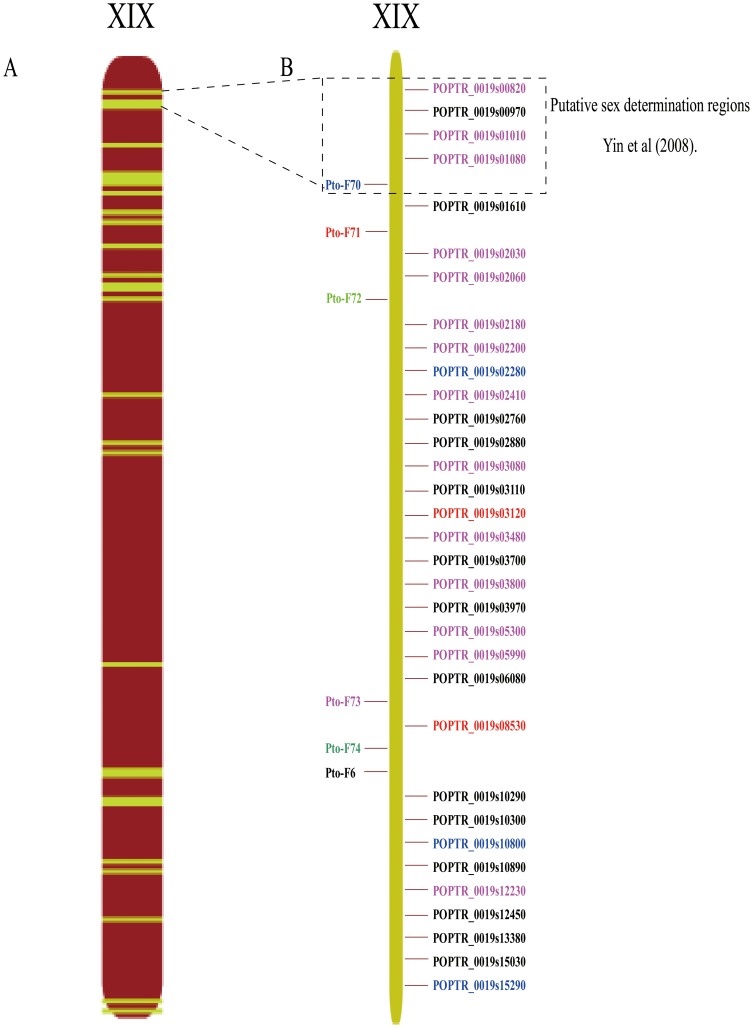
Distribution of novel miRNAs on chromosome XIX. A. 6 novel miRNAs and their targets located on chromosome XIX. B. Schematic diagram of the order of 6 novel miRNAs and their targets on chromosome XIX. Each miRNA and corresponding targets are marked the same color. Putative sex determination region in a 2.45-Mb region at the peritelomeric end of chromosome XIX, based on Yin et al (2008).

It was reported that Chromosome XIX is an incipient sex chromosome in *Populus* [21], [22]. In the present study, 5 novel miRNAs located in chromosome XIX were identified, including Pto-F6, Pto-F70, Pto-F71, Pto-F72 and Pto-F73 (Table 6; Figure 4A). Pto-F6 was expressed both in female and male flower libraries, but expressed at higher levels in female flowers than in male (Table 3). The Pto-70, Pto-71, Pto-72 and Pto-73 female-specific miRNAs were weakly expressed in female flowers (Table 5). A total of 33 targets located on chromosome XIX were identified for these novel miRNAs. Among these, Pto-F6 and Pto-73 were predicted to match 14 targets respectively that are located on chromosome XIX (Table 6). By contrast, no targets were identified for Pto-F72. Putative functions of these targets genes were annotated by BLAST searches against the TAIR, NCBI and JGI protein datasets. The results revealed that target genes are involved in disease resistance, response to auxin stimulus, decreased DNA methylation and membrane transport. Yin et al (2008) indicated that a 2.45 Mb segment in the peritelomeric region of chromosome XIX and is associated with gender determination [21]. In our study, Pto-70 and 4 targets genes were found in sex-determination regions (Figure 4B). Among the 4 target genes, 3 targets were predicted to match Pto-73 miRNAs and one was predicted to match Pto-F6 miRNA.

**Table 6:** Targets of novel *P. tomentosa* miRNAs verified in XIX chromosome of poplar.

miRNA	Target Gene Model^a^	Putative function^b^	Location	TAIR Gene Model^c^
Pto-F6	POPTR_0019s00970	negative regulation of catalytic activity, proteolysis,	Chr19∶737356–739497	AT1G04110
	POPTR_0019s01610	response to auxin stimulus	Chr19∶1429616–1430026	AT5G53590
	POPTR_0019s02760	protein phosphorylation	Chr19∶2561092–2568350	AT1G53430
	POPTR_0019s02880	Pentatricopeptide repeat	Chr19∶2750320–2751951	AT2G34400
	POPTR_0019s03110	Encodes a lipid acyl hydrolase with wide substrate specificity	Chr19∶3122643–3124999	AT2G26560
	POPTR_0019s03700	NB-ARC domain-containing disease resistance protein	Chr19∶3775544–3779105	AT4G27220
	POPTR_0019s03970	NB-ARC domain-containing disease resistance protein	Chr19∶4034540–4038218	AT4G27220
	POPTR_0019s06080	KAR-UP F-box 1 (KUF1); CONTAINS InterPro DOMAIN/s	Chr19∶6589704–6590843	AT1G31350
	POPTR_0019s10290	LRR and NB-ARC domains-containing disease resistance protein	Chr19∶11867256–1870843	AT3G14460
	POPTR_0019s10300	LRR and NB-ARC domains-containing disease resistance protein	Chr19∶11884201–11888700	AT3G14460
	POPTR_0019s10890	similar to expression supported by MPSS	Chr19∶12374345–12375027	AT1G35430
	POPTR_0019s12450	similar to chloroplast precursor (TRX-M1)	Chr19∶13509839–13510150	AT4G03520
	POPTR_0019s13380		Chr19∶14290118–14294196	AT5G08415
	POPTR_0019s15030	similar to decreased DNA methylation 1	Chr19∶15655586–15661763	AT5G66750
Pto-F70	POPTR_0019s02280	member of Myosin-like proteins	Chr19∶2112309–2122774	AT1G54560
	POPTR_0019s10800	Encodes a cyclase	Chr19∶12293003–122983961	AT1G78955
	POPTR_0019s15290	unknown protein	Chr19∶15825895–15830273	AT5G01970
Pto-F71	POPTR_0019s03120	Encodes a lipid acyl hydrolase with wide substrate specificity	Chr19∶3134481–3137841	AT2G26560
	POPTR_0019s08530	ATP phosphoribosyl transferase	Chr19∶10043845–10045971	AT1G09795
Pto-F73	POPTR_0019s00820	ENTH/VHS/GAT family protein	Chr19∶545449–549077	AT5G63640
	POPTR_0019s01010	NB-ARC domain-containing disease resistance protein	Chr19∶774829–779781	AT4G27220
	POPTR_0019s01080	NB-ARC domain-containing disease resistance protein	Chr19∶820958–823839	AT4G27220
	POPTR_0019s02030		Chr19∶1889627–1891189	
	POPTR_0019s02060	LRR and NB-ARC domains-containing disease resistance protein	Chr19∶1922498–1924769	AT4G10780
	POPTR_0019s02180	NB-ARC domain-containing disease resistance protein	Chr19∶2029692–2032970	AT4G27220
	POPTR_0019s02200	Disease resistance protein (CC-NBS-LRR class) family	Chr19∶2058206–2062218	AT1G12290
	POPTR_0019s02410		Chr19∶2215592–2216219	
	POPTR_0019s03080	NB-ARC domain-containing disease resistance protein	Chr19∶3094719–3100126	AT4G27220
	POPTR_0019s03480	similar to phosphoenolpyruvate-carboxylase kinase	Chr19∶3561751–3563659	AT3G04530
	POPTR_0019s03800	similar to SNF2 domain-containing protein	Chr19∶3871132–3874229	AT1G08600
	POPTR_0019s05300	similar to putative disease resistance gene analog NBS-LRR.	Chr19∶5594198–5607481	AT4G27190
	POPTR_0019s05990	integral membrane transporter family protein	Chr19∶6422952–6425657	AT1G64890
	POPTR_0019s12230	similar to GTP-binding protein	Chr19∶13325801–13327862	AT5G60860

Target Gene Model^a^ and Putative function^b^ were derived from Joint Genome Institute (JGI: http://www.jgi.doe.gov/) and (TAIR : http://www.arabidopsis.org).

TAIR Gene Model^c^ were derived from TAIR (TAIR : http://www.arabidopsis.org).

### Target Prediction for Pto-miR156, Pto-miR159, Pto-miR172 and Pto-miR319 and Novel miRNAs in Andromonoecious *P. tomentosa*

**Figure 5:**
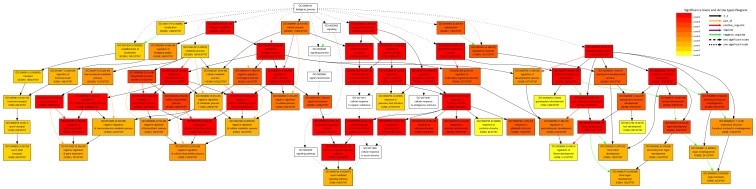
AgriGO analysis of target genes for statistically enriched GO terms in the ‘Biological process’ ontology. Coloring of GO term nodes is proportional to their significance as indicated by the scale.

To understand the functions of sex-specific flower development related miRNAs, the first step is to predict and experimentally validate their targets. Consequently, we predicted 25 target genes for Pto-miR156, Pto-miR159, Pto-miR172 and Pto-miR319 that were different from the previously defined *Populus* miRNA targets (miRBase 19.0), and a total of 464 targets were identified for the 78 novel miRNAs (Table S3). The number of predicted targets varied from 1 to 15 per miRNA. The categorization of female and male-specific miRNA target genes according to biological process is shown in Figure 5. After putative functional annotation by BLAST searches against the TAIR, NCBI and JGI protein datasets, all targets of female and male-specific miRNAs that had a greater than twofold difference in expression between the sexes (Ratio≧2or≦0.5) were identified as candidate genes for gene functional enrichment analysis. All the target genes between the sexes were functionally characterized using enrichment analysis of gene sets, mostly as described by Gene Ontology terms (Figure 5). GO analysis showed that response to hormone stimulus, negative regulation of gene expression, regulation of flower development, and organ formation were significantly enriched terms for biological processes.

### Expression Profiling of miRNAs and Targets in Andromonoecious *P. tomentosa*

**Figure 6:**
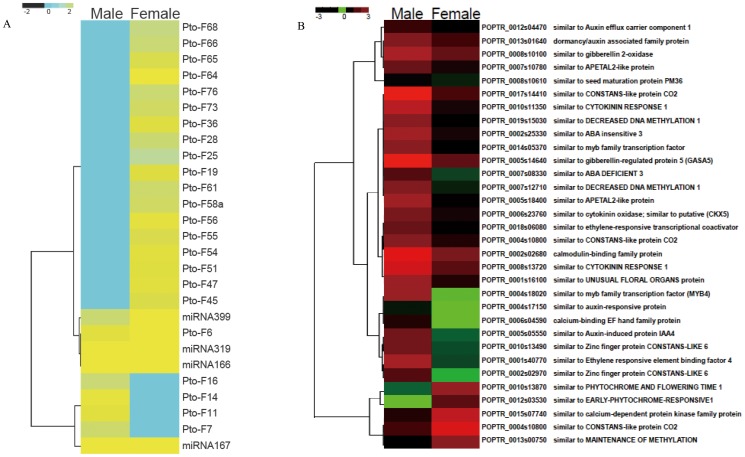
Pearson correlation Coefficient (PCC) heat map representing expression of miRNAs and their targets in andromonoecious poplar flower organs. Red and yellow indicate higher levels of transcriot and green and blue indicate lower levels of transcript. The names of samples are shown at the top. The gene model and gene annotation are shown on the right side. A: Heatmap of conserved and novel miRNAs. B: Heatmap of target genes.

Andromonoecious poplar, an exceptional model system, eliminates confounding effects of genetic background of dioecious plants and thus is a powerful system in which to identify accurate sex-specific factors related to flower development. Therefore, the comparison of the small RNA profiles of female and male flowers of andromonoecious poplar was informative for characterization of functional miRNAs involved in sex-specific flower development. We sequenced 134 conserved miRNA sequences and 78 predicted novel miRNA sequences. 70.1% (94/134) of conserved miRNA sequences were up-regulated in female flower, and only 40 conserved miRNAs were up-regulated in male flower. A total of 104 conserved and 2 novel miRNAs were verified as differentially expressed by greater than two-fold between the two libraries (ratio >2 or <0.5 and p<0.01), including 94 conserved and 2 novel miRNAs that were up-regulated in female flower and 37 conserved and 4 novel miRNAs that were up-regulated in male flower (Table 2 and 3). Moreover, 3 conserved miRNAs (miRNA 397, miRNA 6459 and miRNA6462) and 72 novel miRNAs, including 11 male-specific and 61 female-specific, were expressed in a sex-specific manner (Table 4 and 5). To examine the detailed expression pattern of these potential miRNAs in andromonoecious poplar flowers, the 27 differentially expressed miRNAs, including four conserved and 23 novel miRNAs, were verified by real-time quantitative PCR (Figure 6A). Overall, comparisons of these data correspond to the deep sequencing results, indicating that the miRNA expression levels determined by high-throughput sequencing are reliable.

Base on the results of gene enrichment analysis, 31 putative target genes were selected as candidate genes for gene expression analysis (Table 7 and Figure 6B). The expression of miRNA target genes was detected using real-time quantitative PCR. For most target genes, expression was negatively correlated with the levels of a given miRNA, which is in accordance with the gene silencing function of miRNAs. It should be noted that expression of *CONSTANS-LIKE 6* (*COL6*), the target of PtoF25, was not correlated with the abundance of PtoF25 (Figure 6B), indicating that *COL6* expression might be regulated by other mechanisms.

**Table 7:** Candidate targets of novel and conserved miRNAs in andromonoecious *P. tomentosa*.

miRNA	Cleave sites	Target Gene Model^a^	Putative function^b^	Accession number	TAIR Gene Model
miRNA167	392	POPTR_0013s00750	similar to MAINTENANCE OF METHYLATION	KC477290	AT5G66750
Pto-F6	235	POPTR_0005s14640	similar to gibberellin-responsive protein 5	KC477281	AT3G02380
Pto-F6	305	POPTR_0004s18020	similar to myb family transcription factor (MYB4);	KC477284	AT1G74670
Pto-F6	394	POPTR_0019s15030	similar to DECREASED DNA METHYLATION 1	KC297686	AT1G08060
Pto-F7	193	POPTR_0004s10800	similar to CONSTANS-like protein CO2.	KC477292	AT4G38620
Pto-F11	228	POPTR_0012s03530	similar to EARLY-PHYTOCHROME-RESPONSIVE1;	KC477285	AT1G18330
Pto-F14	336	POPTR_0010s13870	similar to PHYTOCHROME AND FLOWERING TIME 1	KC477286	AT3G60460
Pto-F16	310	POPTR_0015s07740	calcium-dependent protein kinase family protein	KC477266	AT1G68520
Pto-F19	548	POPTR_0002s02970	similar to Zinc finger protein CONSTANS-LIKE 6	KC477293	AT1G25540
Pto-F19	270	POPTR_0014s05370	similar to myb family transcription factor	KC297692	AT1G75540
Pto-F25	139	POPTR_0010s13490	similar to Zinc finger protein CONSTANS-LIKE 6	KC477294	AT1G19870
Pto-F28	293	POPTR_0002s02680	calmodulin-binding family protein	KC477283	AT4G36920
Pto-F36	283	POPTR_0005s18400	similar to APETAL2-like protein	KC477268	AT3G10300
Pto-F45	481	POPTR_0006s04590	calcium-binding EF hand family protein	KC477282	AT3G15210
Pto-F47	125	POPTR_0001s40770	similar to Ethylene responsive element binding factor 4	KC477274	AT4G34760
Pto-F47	99	POPTR_0004s17150	similar to auxin-responsive protein	KC477279	AT4G36920
Pto-F51	815	POPTR_0007s10780	similar to APETAL2-like protein	KC477295	AT3G16990
Pto-F54	224	POPTR_0008s10610	similar to seed maturation protein PM36	KC477289	AT1G74740
Pto-F54	180	POPTR_0001s16100	similar to UNUSUAL FLORAL ORGANS protein	KC477287	AT1G30950
Pto-F55	321	POPTR_0007s08330	similar to ABA DEFICIENT 3	KC477272	AT5G66750
Pto-F56	513	POPTR_0010s11350	similar to CYTOKININ RESPONSE 1	KC477269	AT2G01830
Pto-F56	167	POPTR_0007s12710	similar to DECREASED DNA METHYLATION 1	KC477291	AT1G16540
Pto-F58a	311	POPTR_0008s13720	similar to CYTOKININ RESPONSE 1	KC477270	AT2G01830
Pto-F64	168	POPTR_0018s06080	similar to ethylene-responsive transcriptional coactivator	KC477275	AT3G24500
Pto-F65	152	POPTR_0002s25330	similar to ABA insensitive 3	KC477273	AT3G02380
Pto-F66	71	POPTR_0004s10800	similar to CONSTANS-like protein 1	KC477292	AT3G24650
Pto-F66	187	POPTR_0017s14410	similar to CONSTANS-like protein 1	KC477267	AT5G43700
Pto-F68	253	POPTR_0012s04470	similar to Auxin efflux carrier component 1	KC477277	AT3G02380
Pto-F68	166	POPTR_0005s05550	similar to Auxin-induced protein IAA4	KC477278	AT1G73590
Pto-F68	89	POPTR_0008s10100	similar to gibberellin 2-oxidase	KC477280	AT1G47990
Pto-F73	421	POPTR_0013s01640	dormancy/auxin associated family protein	KC477276	AT5G21482
Pto-F76	221	POPTR_0006s23760	similar to cytokinin oxidase; similar to putative (CKX5)	KC477271	AT1G56220

Target Gene Model^a^ and Putative function^b^ were derived from Joint Genome Institute (JGI: http://www.jgi.doe.gov/) and (TAIR : http://www.arabidopsis.org).

### A Possible Functional Network of Sex-specific Flower Development Related miRNAs in Andromonoecious *P. tomentosa*

**Figure 7:**
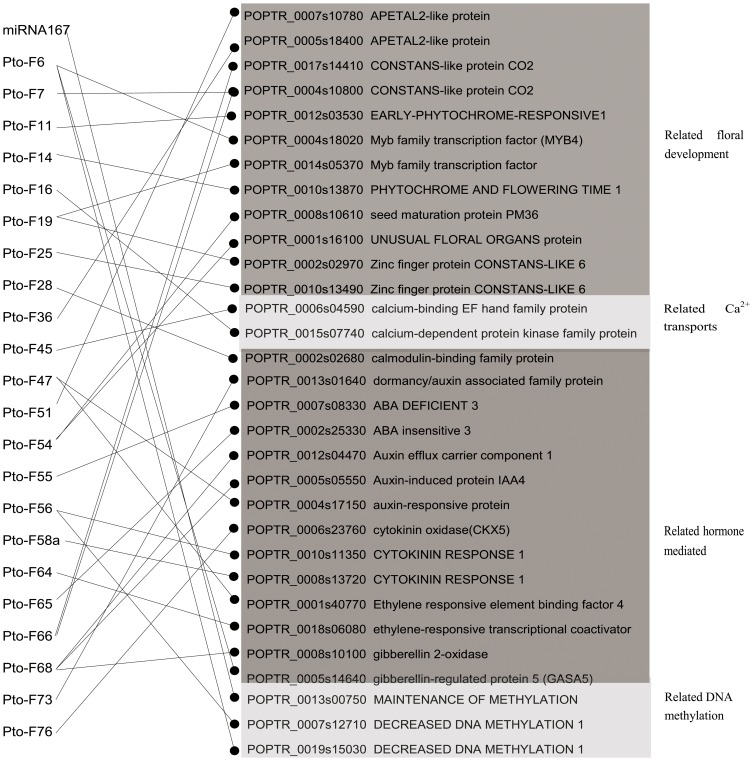
A possible functional network of flower development related miRNAs in andromonoecious *P. tomentosa*. Black line represent repression of target gene expression.

The idea that miRNAs could play important roles in flower development has been suggested in previous studies. However, no systematic investigation has been conducted into how flower development may be regulated via miRNA-mediated processes. In the present study, a possible functional network, which connects flower development related miRNAs with their targets were first reported (Table 7 and Figure 7). Base on the gene functional annotation, all target genes were divided into four subgroups as shown in Figure 7.

The first subgroup related to floral development was regulated by 9 miRNAs including Pto-F6, Pto-F11, Pto-F14, Pto-F19, Pto-25, Pto-36, Pto-F51, Pto-F54 and Pto-F66. Four members of the *CONSTANS-LIKE* gene family were negatively regulated by three female-specific miRNAs including Pto-F19, Pto-F25 and Pto-F66. Of these, Pto-F66 was found to target 2 members of the *CONSTANS-LIKE2* gene family. Also, *EARLY-PHYTOCHROME-RESPONSIVE1* (*EPR1*) and *PHYTOCHROME AND FLOWERING TIME 1* (*PFT1*) were negatively regulated by two male-specific miRNAs, Pto-F11 and Pto-F14, respectively. In our study, we detected two novel miRNAs, Pto-F36 and Pto-51, targeting *APETALA 2* (*AP2*). Female flower specific Pto-F54 was found to target two genes, *UNUSUAL FLORAL ORGANS* (*UFO*) and *SEED MATURATION PROTEIN* (*PM36*).

Three miRNAs are involved in regulation of the second subgroup, genes that relate to Ca^2+^ transport. Two female flower specific miRNAs, Pto-F28 and Pto-F45, were found to be negative factors for the expression of *CALMODULIN-BINDING FAMILY PROTEIN* and *CALCIUM-BINDING EF HAND FAMILY PROTEIN*. By contrast, *CALCIUM-DEPENDENT PROTEIN KINASE FAMILY PROTEIN* (*CDPK*) gene expression was repressed by Pto-F16, a male-specific miRNA.

In the third subgroup, there were 10 miRNAs involved in hormone related gene regulation. All of these miRNAs were female flower specific, except Pto-F6, which had expression significantly higher in female flower than in male. Pto-F68 has three target genes, including *AUXIN EFFLUX CARRIER COMPONENT 1*, *AUXIN-INDUCED PROTEIN* (*IAA4*) and *GIBBERELLIN 2-OXIDASE*. By contrast, *ETHYLENE RESPONSIVE ELEMENT BINDING FACTOR 4* (*EREBF4*) and *AUXIN-RESPONSIVE PROTEIN* (*SAUR29*) were targeted by the same novel miRNA, Pto-F47. Two genes related to ABA, *ABA DEFICIENT 3* (*ABA3*) and *ABA INSENSITIVE 3* (*ABI3*), were targeted by female flower specific miRNAs Pto-F55 and Pto-F65, respectively.

In the last subgroup, genes that relate to DNA methylation were regulated by 3 miRNAs. *MAINTENANCE OF METHYLATION1* (*MET1*) was negatively regulated by miRNA167, which had expression significant higher in male flower than in female. By contrast, two members of *DECREASED DNA METHYLATION 1* (*DDM1*) were negatively regulated by Pto-F6, which had higher expression in female flower and by female flower specific Pto-F56, respectively.

## Discussion

### Overview of the Deep Sequencing Datasets

Although an increasing number of studies about miRNAs and their expression patterns from different tissues have been reported, there are still few studies on sex-specific miRNAs between female and male flowers of poplar. In this study, andromonoecious poplar, an exceptional model system, was used to eliminate confounding effects of genetic background of dioecious plants. We generated sRNA libraries from female and male flower of andromonoecious poplar and obtained over 40 million reads of 18–30 nt per library, substantially increasing the available data on poplar flower sRNAs [23], [24]. Mapping analysis of unique sRNAs revealed that over 60% of the identified sRNAs were previously unknown, suggesting that even more sRNAs remain to be identified. The size distribution of small RNAs indicated that the numbers of 21-nt sRNAs were significantly higher than 24-nt sRNAs in female flower, suggesting that female flower development may require more sRNAs in this size class. By contrast, the numbers of 21-nt sRNAs and 24-nt sRNAs were not significantly different in male flower libraries. The 24-nt sRNAs mainly comprised siRNAs associated with repeats and transposons [25], [26]. 24-nt sRNA numbers in male flower were significantly higher than in female, suggesting that a number of siRNAs related to repeats and transposons are involved in male flower development.

### Conserved miRNAs Expressed in Flower

We detected dramatically different expression levels among different members of the same miRNA family, suggesting that expression of conserved miRNAs varies in andromonoecious *P. tomentosa*. These conserved miRNAs have been identified in multiple flowering pathways. For example, three miRNA families (miR172, miR159/miR319 and miR156) are involved in flowering-time regulation [27]. In this study, miRNA159 and miRNA319 expression in female flower was 38.8-fold and 51.42-fold higher, respectively, than in male flower. miRNA172 was weakly expressed in female and male flower, with no significant difference. By contrast, miRNA156 was expressed in male flower at higher levels than in female flower. This result suggested that different miRNAs are involved in female and male flowering time regulation. miRNA159 and miRNA319 also have been reported to act with miRNA164 and miRNA167 in specifying particular cell types during the later stages of flower development [28]. The present study revealed that miRNA164 was not expressed in female or male flower, indicating miRNA164 might show stage-specific expression in flower development. miRNA167 was up-regulated dramatically in male flower, indicating that it may play an important role in specifying male flower cell types. miRNA156, miRNA157 and miRNA172 may be components of a regulatory pathway mediating the transition between the vegetative and reproductive phases in plants [28]. miRNA166, targeting *APETALA2* and type III homeodomain-leucine zipper (HD-Zip) family genes, was dramatically up-regulated in female flower, suggesting *AP2* and HD-ZiP genes might be negatively regulated in female flower [29]. miR167 and miR160 are thought to control transcription in response to the phytohormone auxin by targeting mRNAs coding for ARF DNA-binding proteins [30], [31]. These miRNAs were detected in this study as having opposite expression patterns between female and male flower, suggesting that two miRNAs involved in regulation of auxin responses in female flower are different compare with in male flower.

### Novel miRNAs Displayed Different Expression Patterns between Female and Male Flower

The 78 novel miRNAs identified here spanned a size range of 20–22 nt, and the majority started with a 5′ U, consistent with results in Japanese apricot [32]. The majority of novel miRNAs showed relatively low expression levels, which was consistent with previous studies predicting that novel miRNAs are often expressed at lower levels than conserved miRNAs [33]. The miRNA*s were generally less abundant than the corresponding mature miRNAs. It has been reported that miRNA*s have important functions through regulation of the miRNA precursors themselves when miRNA*s were more abundant than their corresponding miRNAs [34]. miRNA*s identified were used as extremely strict criteria to identify novel miRNAs [35]. In our study, only 8 unique miRNAs were detected with complementary miRNA*s. Most novel miRNAs do not have identified miRNAs*, likely because of the depth of sequencing, which results in a low probability of identifying low-frequency miRNA*s. The numbers of female-specific novel miRNAs was 6-fold higher than male-specific novel miRNAs, suggesting that abundant novel miRNAs are involved in female flower development. A large proportion of the novel sRNAs identified in andromonoecious *P. tomentosa* did not have perfect matches in different genome databases, providing more evidence to support our conclusion that these novel miRNAs might be poplar-specific and were first reported in present study. Among all of the novel miRNAs, 11 miRNAs were found with perfect matches in other species, suggesting that some of these newly identified miRNAs may be broadly conserved within angiosperms.

### Novel miRNAs Located in Sex Determination Regions

Although studies on determination of gender indicate that dioecy in *Populus* is genetically controlled, the precise gender-determining systems remain unclear. Based on genetic mapping results, a gender determination locus mapped to different positions on chromosome XIX, depending upon the *Populus* species [36]–[38]. Klevebring et al. (2009) found that the proposed sex-determining peritelomeric region of chromosome XIX showed a distinctive pattern of sRNA occurrence that differed significantly from the rest of the genome [39]. In our study, 5 novel miRNAs and 33 targets genes were located on chromosome XIX, indicating that these miRNAs might be involved in sex determination of poplar. The annotated results revealed that over one-third of targets were annotated as disease resistance genes. It has been reported that a NBS–LRR gene supercluster was located on *Populus* chromosome XIX. These results are consistent with the hypothesis that resistance to a floral pathogen and regulation of gender determination coevolved in a region of the genome that experiences reduced recombination [22]. Interestingly, Pto-F6 was predicted to match a target that encodes DNA (cytosine-5) -methyltransferase implying that DNA methylation might act in flower development.

### miRNA Targets and Sub-network Analysis

To better understand the functions of identified miRNAs, we sought to identify potential targets in andromonoecious *P. tomentosa* using computational analyses. Many targets of conserved miRNAs have been predicted and their functions annotated [40], [41]. Integrating target genes and miRNA expression data identified sub-networks for further investigation. *COL1* encodes a zinc finger protein belonging to the first groupings of the flowering-time gene *CONSTANS* (*CO*) [42]. Over-expression of *COL1* can shorten the period of two distinct circadian rhythms by affecting a light input pathway [43]. In our study, *COL1* was targeted by female flower specific Pto-F66, which was expressed in female flowers suggesting that the novel miRNA Pto-F66 likely plays a negative role in the light input pathway in female flower. By contrast, *COL6* is part of the third groupings of *CO*, and is a target of Pto-F19 and Pto-F25. However, these two miRNAs were not correlated with *COL6* transcript abundance, indicating that *COL6* expression might be regulated by other mechanisms. The target of male specific miRNA Pto-F11, *EPR1* encodes a nuclear-localized MYB protein showing rapid induction in response to red light irradiation. *EPR1* was significantly down-regulated in male flower, suggesting that this primary phytochrome-responsive factor is likely repressed by the male flower specific novel miRNA Pto-F11 [44]. Another gene involved in the photoperiodic pathway, *PFT1* transcript abundances were significantly decreased in male flower. *PFT1* is a target of PtoF14 and functions downstream of phyB to regulate the expression of *FLOWERING LOCUS T* (*FT*), providing evidence that Pto-F14 might be involved in regulation of flowering time by affecting the light quality pathway [45].

The *AP2* gene family contains 144 members encoding at least one AP2 DNA-binding domain and the varied biological functions of AP2 family members range from development to stress and defense responses [46]–[48]. Conserved miRNA172 has been identified as an important factor regulating AP2 transcription factors [17]. In our study, miRNA172 was equally expressed in female and male flower, but two novel miRNAs, Pto-F36 and Pto-F51, were detected with female-specific expression. *AP2* transcript abundance decreased in female flower, providing evidence that these two miRNAs act in regulation of *AP2*. *UFO* encodes an F-box protein required for the determination of floral-organ and floral-meristem identity [49]. *PM36* is involved in reproductive system development. These genes were targeted by the female-specific novel miRNA Pto-F54 and *UFO* and *PM36* expression were down-regulated in female flower, indicating the broad biological functions of Pto-F54. Calcium is a ubiquitous second messenger in plant signal transduction cascades [50]. Three novel miRNAs were identified as related to Ca^2+^ transport, including one male flower specific and two female flower specific miRNAs, suggesting that Pto-F16, Pto-28 and Pto-45 are likely play different roles in regulating calcium transport in female and male flower development.

Hormones play a central role in the coordination of internal developmental processes with external environmental signals [51]. A series of genes related to phytohormone synthesis and metabolism are differentially expressed between female and male during flower development (unpublished data). In our study, 13 genes related to five important phytohormones were found to be targeted by 12 novel miRNAs, suggesting that these miRNAs act broadly in regulation of phytohormone synthesis and metabolism. Previous work reported that auxin is necessary for GA-mediated control of root growth, and that attenuation of auxin transport or signaling delays the GA-induced disappearance of RGA (repressor of gal-3) [52]. Among these miRNAs, Pto-F68 targets three genes involved in auxin transport and gibberellin synthesis suggesting that Pto-F68 may regulate the interaction of auxin and gibberellin. Also, a series of physiological studies indicate that ethylene and auxin interact in a number of different ways depending on the cell type, developmental stage, and environmental conditions [51]. *EREBF4* and *SAUR2* expression were downregulated in female flower and targeted by Pto-F47 miRNA. This result may provide a clue that deepens our understanding of the important regulatory functions of miRNA Pto-F47 in phytohormone interactions. Poplar male flowers abscise earlier than female flowers, suggesting the ABA accumulation and regulation of related genes might be different between female and male flowers. *ABA3* encodes an enzyme that catalyzes the generation of the sulfurylated form of MoCo, a cofactor required by aldehyde oxidase, which functions in the last step of ABA biosynthesis in plants [53]. *ABI3* encodes a DNA binding factor targeted to promoters responsive to ABA and auxin [54]. These two genes related to ABA biosynthesis and responses are targeted by novel miRNAs Pto-F55 and Pto-F65, which have female flower specific expression, suggesting these miRNAs have negative roles in flower abscission through regulation of ABA metabolism.

*MET1*, encoding DNA (cytosine-5) –methyltransferase, which maintains CG methylation, was down-regulated in male flower [55]. *DDM1*, encoding a chromatin remodeling complex with ATPase activity that can remodel nucleosomes, was up-regulated in male flower [56]. *MET1* and *DDM1* play opposite roles in the regulation of DNA methylation levels; therefore, the different expression of these genes in female and male flowers might alter DNA methylation levels, which are higher in female flower than male (unpublished). Our study indicated that miRNA167, Pto-F6 and Pto-F56 might play a positive regulatory role in maintaining DNA methylation levels of female flower through regulation of *MET 1* and *DDM1*.

## Materials and Methods

### Plant Materials

Four-hundred and sixty unrelated individuals, representing almost the whole geographic distribution of *P. tomentosa*, were collected in the national nursery of Guan Xian County (Shandong Province, China). After observation of flower morphology, three 29-year-old unrelated andromonoecious clones (‘2–14’, ‘3605’, and ‘5103’) were found.

All three individuals were used in our study. Sampling that female and male flowers were dissected from the andromonoecious flowers was performed at the last phase of flower development, before pollination. All materials were collected from poplar individuals and immediately frozen in liquid nitrogen, and stored at −80°C for isolation of RNA. To eliminate genetic backgroud, the two bulks were made by mixing equal amounts of RNA extracted from three andromonoecious poplar female and male flower, respectively.

This study was carried out in strict accordance with the recommendations in the Guide for Observational and field studies (http://www.plosone.org/static/publication **)**. All necessary permits were obtained for the described field studies. The sampling of all individuals of *P*. *tomentosa* was approved by Youhui Zhang, director of National Garden of *P*. *tomentosa* in Guan Xian County, Shandong Province.

### Total RNA Isolation, sRNA Library Construction, and Solexa Sequencing

Total RNA was isolated from female and male floral tissue by a modified CTAB method [57] with isopropanol instead of lithium chloride for RNA precipitation. Extracted RNA was used for sRNA library construction. Small RNAs were sequenced using an Illumina HiSeq 2000 at the Shanghai Bio Institute.

Sequence data from this article have been deposited in the GenBank Data Library under the accession Nos: KC477266–KC477295.

### Bioinformatic Analyses of Sequencing Data and Target Prediction of miRNAs

Clean reads were screened from raw reads by removing contaminating reads, including sequences with adapters, without the insert tag, with poly-A tails, shorter than 18 nt, or longer than 30 nt. Clean reads were then aligned with the *Populus* genome (http://www.phytozome.net/poplar) using SOAP [58], with no mismatches allowed. Small RNA tags matching exons and introns of mRNAs were excluded from further analysis. Tags matching non-coding RNAs, including rRNAs, scRNA, tRNAs, snRNAs, and snoRNAs, deposited at the Rfam (http://www.sanger.ac.uk/Software/Rfam) and NCBI GenBank databases (http://www.ncbi.nlm.nih.gov/blast/Blast.cgi) [59] were also excluded from further analysis. The remaining unannotated sRNAs were searched against miRBase 19.0 with a maximum of two mismatches allowed [20] to identify conserved miRNAs in *P*. *beijingensis* and then the resulting sequences were screened for the presence of the characteristic hairpin structures using the program RNAfold (http://www.tbi.univie.ac.at/ivo/RNA/ViennaRNA-1.8.1.tar.gz) [60]. We used the prediction software Mireap (https://sourceforge.net/projects/mireap/) to predict novel miRNAs by exploring the secondary structures, DL1 cleavage sites, and minimum free energies of the unannotated sRNA tags that could be mapped to the *Populus* genome. Basic criteria [35] were used for selecting the potential novel miRNAs. Target genes were predicted in accordance with previous research [61], [62] but with higher stringency; only three mismatches were allowed in the miRNA/target duplex. Target searches were performed using *Populus* transcripts deposited in the Phytozome v7.0 (http://www.phytozome. net/) and Pfam databases (http://pfam.sanger.ac.uk/), and the results were used to annotate the functions of potential targets. We compared miRNA expression between the two libraries (control and treatment) to determine which miRNAs were differentially expressed. We normalized the expression levels of miRNAs within each library to get the expression of transcripts per million.

### 5′-RACE

RNA Ligase-Mediated 5′-RACE (RLM-RACE) was performed with the First Choice RLM-RACE Kit (Ambion) according to the manufacturer’s instructions, with slight modifications. Briefly, 10**µg of total RNA was directly ligated to the 5′ adaptor followed by reverse transcription with an oligo (dT) primer. PCR was performed with 5′ adaptor primers and 3′ gene-specific primers (Table S4) using cDNA as the template. The RACE products were gel-purified, cloned, and sequenced.

### qRT-PCR

Small RNAs (<200 nt) were isolated using the mirVana miRNA Isolation Kit (Ambion, USA) following the manufacturer’s instructions. Quantitative real-time PCR (qRT-PCR) was carried out as previously described [63] in an ABI PRISM 7500 Fast Real-time PCR System (Ambion, USA) using the SYBR Premix Ex TaqTM Kit (TaKaRa, Japan). The reactions were carried out in a 20 µl volume containing 2 µl of diluted cDNA, 200 nM of each primer, and 19 PCR Master Mix with the following conditions: 95°C for 30 s, and 45 cycles of 95°C for 5 s, 58°C for 15 s, and 72°C for 20 s. Then, a thermal denaturing cycle of 95°C for 15 s and 60°C for 1 min was applied to determine the dissociation curves, which were used to verify the specificity of PCR amplifications. All reactions were run in triplicate for each sample. Twelve miRNAs, including six conserved and six novel miRNAs were validated, and 5.8S rRNA was selected as a reference gene for normalization [41] (Table S5 and Table S6).

### Network Analysis

The details of sex-specific flower development related miRNAs functional network methods were done according to previously described [64]. Comparative miRNAome analysis combined with realtime quantitative PCR and RACE–PCR revealed 23 miRNAs expressed in andromonoecious poplar flower and each of them has functional targets. Negative correlation between the miRNA and target expression profiles was validated using real-time quantitative PCR. These results indicated that miRNA-mediated regulation does occur in flower development and that these miRNAs with dynamic changes ultimately down-regulate their targets. All miRNA and functional targets were connected for network analysis.

## Supporting Information

Figure S1 **Morphological and microscopic observation of andromonoecious poplar.** A:Morphological characteristics of andromonoecious poplar flowers. B Pollen tubes stained with aniline blue, showing that pollen viability is high and that pollen grows well during selfing. Pt pollen tube, Sti stigma, Pg pollen grain.
(TIF)

Figure S2 **Mature and precursor sequences and the predicted stem-loop structures of novel miRNAs from andromonoecious poplar flower tissue.** The mature miRNAs are in green, and the miRNA*s are in red.
(TIF)

Table S1 134 loci of 38 unique miRNAs.
(XLS)

Table S2 11 poplar novel miRNAs were detected in other species.
(DOC)

Table S3 miRNAs and their targets.
(XLS)

Table S4 Primers for 5′ RACE mapping of miRNA cleavage sites.
(DOC)

Table S5 Real-time PCR primer sequences for candidate miRNAs.
(DOC)

Table S6 Real-time PCR primer sequences for candidate target genes.
(DOC)
