# Supplementary figures and images for: Sexual Dimorphism Floral MicroRNA Profiling and Target Gene Expression in Andromonoecious Poplar (Populus tomentosa)
Source: PLoS One. 2013 May 7;8(5):e62681. doi: 10.1371/journal.pone.0062681 (PMC3646847; doi:10.1371/journal.pone.0062681)

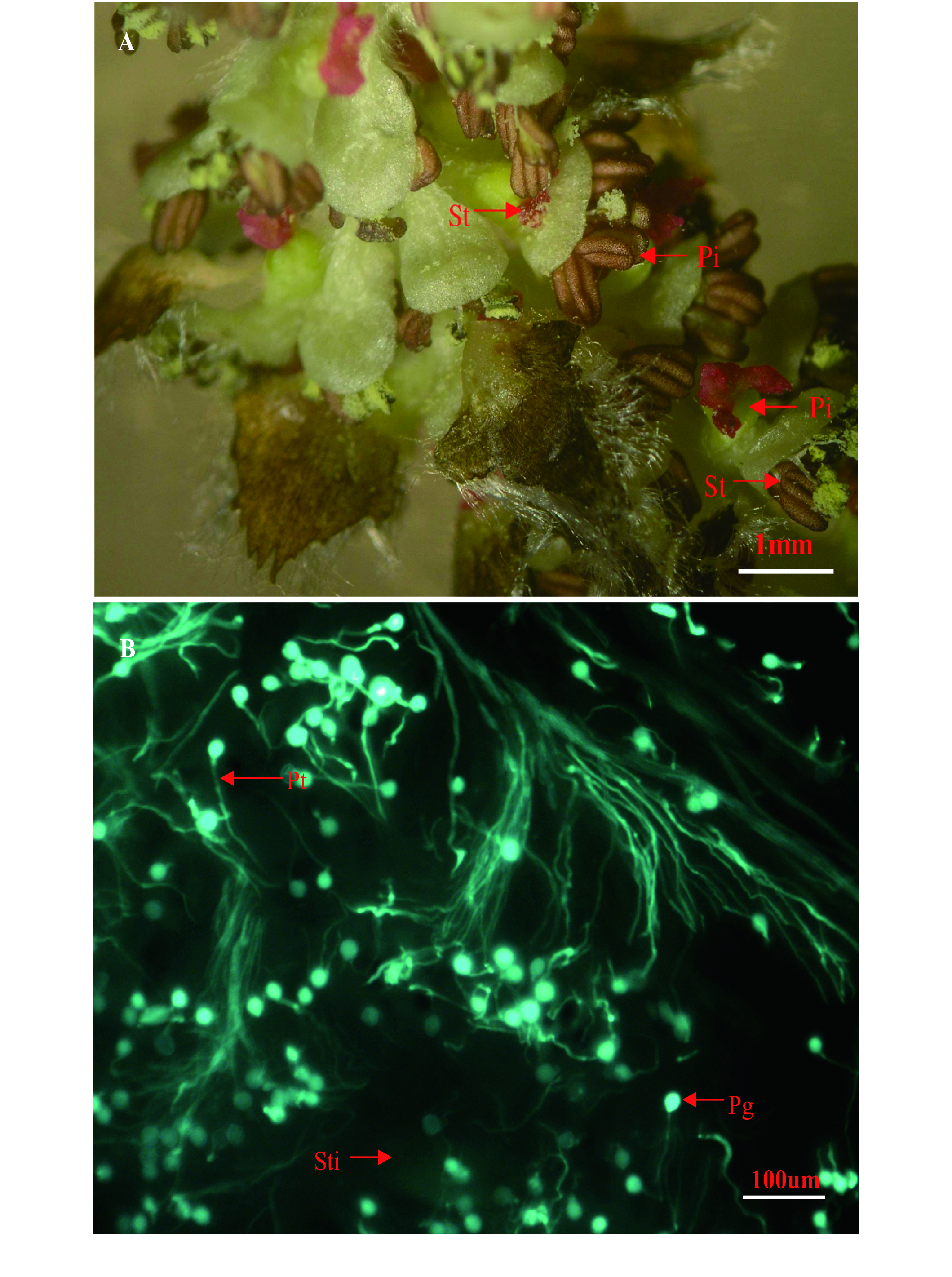

Supplement: Figure S1 — Morphological and microscopic observation of andromonoecious poplar. A:Morphological characteristics of andromonoecious poplar flowers. B Pollen tubes stained with aniline blue, showing that pollen viability is high and that pollen grows well during selfing. Pt pollen tube, Sti stigma, Pg pollen grain. (TIF) [file pone.0062681.s001.tif]

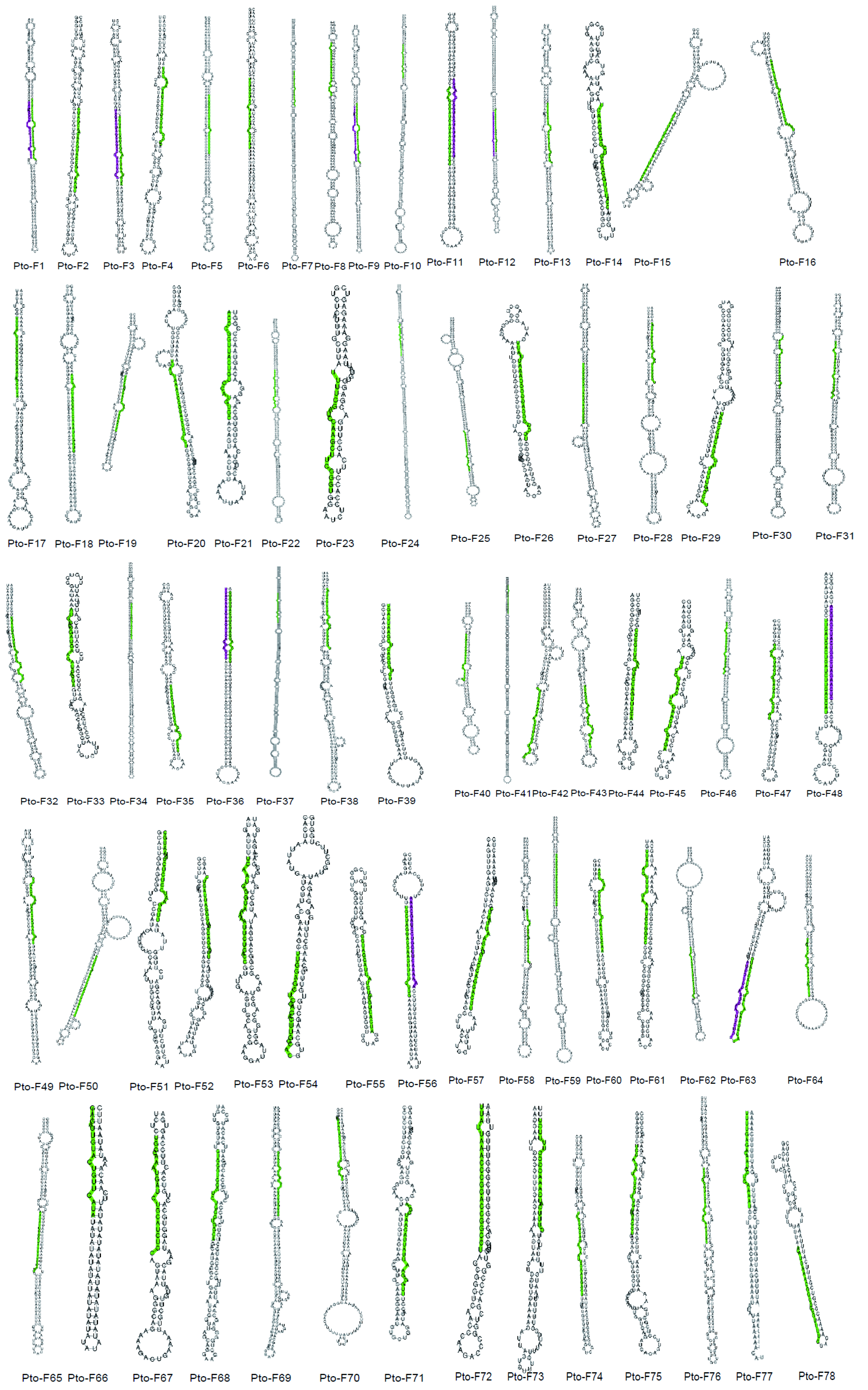

Supplement: Figure S2 — Mature and precursor sequences and the predicted stem-loop structures of novel miRNAs from andromonoecious poplar flower tissue. The mature miRNAs are in green, and the miRNA*s are in red. (TIF) [file pone.0062681.s002.tif]
