# Supplementary material for: Sexual Dimorphism Floral MicroRNA Profiling and Target Gene Expression in Andromonoecious Poplar (Populus tomentosa)
Source: PLoS One. 2013 May 7;8(5):e62681. doi: 10.1371/journal.pone.0062681 (PMC3646847; doi:10.1371/journal.pone.0062681)
Supplement: Table S2 — 11 poplar novel miRNAs were detected in other species. (DOC) [file pone.0062681.s004.doc]

**Table S2.** 11 poplar novel miRNAs were detected in other species

|  | *Arabidopsis thaliana* | *Brassica rapa* | *Citrus sinensis* | *Medcago truncatula* | *Oryza sativa* | *Ricinus comunis* | *Prunus persica* | *Vitis vinifera* | *Zea mays* |
| --- | --- | --- | --- | --- | --- | --- | --- | --- | --- |
| Pto-F7 | **●** |  |  |  |  |  |  |  |  |
| Pto-F9 | **●** |  |  |  |  |  |  |  |  |
| Pto-F12 |  |  |  | **●** |  |  |  |  |  |
| Pto-F14 |  |  |  |  |  |  |  | **●** |  |
| Pto-F15 |  |  |  |  |  |  |  |  | **●** |
| Pto-F17 |  |  |  |  |  |  |  | **●** |  |
| Pto-F33 | **●** |  |  |  |  |  |  | **●** |  |
| Pto-F40 |  |  |  |  |  | **●** |  |  |  |
| Pto-F50 |  |  |  |  |  |  |  | **●** |  |
| Pto-F63 | **●** |  |  | **●** | **●** |  |  |  | **●** |
| Pto-F65 | **●** | **●** |  | **●** |  |  |  |  |  |
