# Supplementary material for: Sexual Dimorphism Floral MicroRNA Profiling and Target Gene Expression in Andromonoecious Poplar (Populus tomentosa)
Source: PLoS One. 2013 May 7;8(5):e62681. doi: 10.1371/journal.pone.0062681 (PMC3646847; doi:10.1371/journal.pone.0062681)
Supplement: Table S4 — Primers for 5′ RACE mapping of miRNA cleavage sites. (DOC) [file pone.0062681.s006.doc]

**Table S4.** Primers for 5′ RACE mapping of miRNA cleavage sites

| Targets gene | Primer sequence(5'-3') |
| --- | --- |
| POPTR_0013s00750outer primer | CTAGTTCTTCTGGTTCAAAG |
| POPTR_0013s00750inner primer | GTCTTGAGAAGCAGACCCCTACGG |
| POPTR_0005s14640outer primer | CAAGGAAGGAAGACCCAAGTG |
| POPTR_0005s14640inner primer | TGCTCAGCAACATCACACAAGAAGCC |
| POPTR_0004s18020outer primer | TGAGCTCATTATCAAACTCCATAG |
| POPTR_0004s18020inner primer | GCGCATGGACTAAGGAAGAAGATGAT |
| POPTR_0019s15030outer primer | CTGCTGGAAAAGATTGATCA |
| POPTR_0019s15030inner primer | TCCCCCTTAAAATGAAGGAGCAA |
| POPTR_0004s10800outer primer | CGGTTCCCCAGTAGGGCCTGCAGC |
| POPTR_0004s10800inner primer | GCGCGTATCGGTGTGCGAGGCGTGTG |
| POPTR_0012s03530outer primer | TTCATTTGGGAGTGATAATGTG |
| POPTR_0012s03530inner primer | GCAAATGGAAGGCACGAATCTGAACT |
| POPTR_0010s13870outer primer | GTTGAGCTCTCAATTGTCAC |
| POPTR_0010s13870inner primer | GGCCGTTGAAGGCACTGCTGCCATG |
| POPTR_0015s07740outer primer | GTGGCTAGAGGGCATTATAGTG |
| POPTR_0015s07740inner primer | CCGCCCTCGTATCTCTGACAAGTAC |
| POPTR_0002s02970outer primer | CTCTGTCACCAACAGTGGTGGTG |
| POPTR_0002s02970inner primer | GAAACGGGCTTTCTTGTTCTGTCAAC |
| POPTR_0014s05370outer primer | CGATGCTGTATTCATCTTCGAG |
| POPTR_0014s05370inner primer | CCTTCTACCCTTCGCGATTTTAACG |
| POPTR_0010s13490outer primer | TCAACAACAGTCTCGAATGAAGC |
| POPTR_0010s13490inner primer | GGCCAACGCTGTTGGTGGCAGAACTGC |
| POPTR_0002s02680outer primer | CCTTCTTCTGTGAACAAGGAG |
| POPTR_0002s02680inner primer | CCTGCGGTAGCGATTCTGCTGACAGAG |
| POPTR_0005s18400outer primer | CATAGCATAAGCTTCAGCAG |
| POPTR_0005s18400inner primer | TGGATCCGTGTCGAATTCCAGCTC |
| POPTR_0006s04590outer primer | CCGGCACCAAGCCACCGAAG |
| POPTR_0006s04590inner primer | GGTTACGGCCAACCGCAACAACCGCA |
| POPTR_0001s40770outer primer | GGATTATCACTTGATCTTGAT |
| POPTR_0001s40770inner primer | AGTATAGCGAATAATATTAACATGT |
| POPTR_0004s17150outer primer | ATGGCTTAAGATGAAGCTAC |
| POPTR_0004s17150inner primer | GAGCCACCCTGAGTTTCAATCCTTG |
| POPTR_0007s10780outer primer | CAGAAGCTCTCAATACCGCG |
| POPTR_0007s10780inner primer | TTCTCGGTGCCTTATGATCAATACTC |
| POPTR_0008s10610outer primer | TCTCCTCTTTCAAGAGATGG |
| POPTR_0008s10610inner primer | GGATCCTGTAGAGAAGAATGGTGTG |
| POPTR_0001s16100outer primer | GGGTTTGATTGTGTTGGACA |
| POPTR_0001s16100inner primer | GTGTTGATGACAGATTTTACTGCATG |
| POPTR_0007s08330outer primer | GTTACAAATGGAAGAAATCT |
| POPTR_0007s08330inner primer | ATCCAAACGGTCCAAAATCTATCG |
| POPTR_0010s11350outer primer | GCTGAGTACACTGCTAGAAC |
| POPTR_0010s11350inner primer | GCCATTCTTGTCTCCACTTTTCATTA |
| POPTR_0007s12710outer primer | CGAGATTGGATGACCTCTTAAC |
| POPTR_0007s12710inner primer | GATCAAAGAGGAAGTGAAATTGGAGG |
| POPTR_0008s13720outer primer | AGGCCACTGTTGAGTGGCGTAG |
| POPTR_0008s13720inner primer | CTAGCCATTCTTGTCTCCACTTTTC |
| POPTR_0018s06080outer primer | TCCGAGCTTCAATCAATCGA |
| POPTR_0018s06080inner primer | GCCAAGATGGAGAGAGTGCTAGGCG |
| POPTR_0002s25330outer primer | AGGCGCATGAGAGAATCATC |
| POPTR_0002s25330inner primer | CTGGCCATGGTGTTTTTAGACTGG |
| POPTR_0017s14410outer primer | GTTGTGCCGATTCAGTATG |
| POPTR_0017s14410inner primer | TCGGCATACCTATGTGCCGGTTGT |
| POPTR_0012s04470outer primer | GCTCAACAGAAAGCTGAAGAT |
| POPTR_0012s04470inner primer | CTACAAAAGATCATAGTTTTAGTAG |
| POPTR_0005s05550outer primer | GCGATTGGATGTTGGTTGGA |
| POPTR_0005s05550inner primer | GGATATCCAGAGCTCCTCGAGGTCGT |
| POPTR_0008s10100outer primer | CTTAATATCAACCCTCTCTC |
| POPTR_0008s10100inner primer | GCATCTCCAACTAAACTTCACAGTG |
| POPTR_0013s01640outer primer | GCTGGTTCTACTCCTCCGGT |
| POPTR_0013s01640inner primer | CTGATGGTAGTGGTGGTAGCGT |
| POPTR_0006s23760outer primer | GCAGAGTTGGATGTTGTTAC |
| POPTR_0006s23760inner primer | ACATATGTCACTCTCTAGACCTCCA |
| 5' RACE Adapter | 5'-GCUGAUGGCGAUGAAUGAACACUGCGUUUGCUGGCUUUGAUGAAA-3' |
| 5' RACE Outer Primer | 5'-GCTGATGGCGATGAATGAACACTG-3' |
| 5' RACE Inner Primer | 5'-CGCGGATCCGAACACTGCGTTTGCTGGCTTTGATG-3' |
