# Supplementary material for: Sexual Dimorphism Floral MicroRNA Profiling and Target Gene Expression in Andromonoecious Poplar (Populus tomentosa)
Source: PLoS One. 2013 May 7;8(5):e62681. doi: 10.1371/journal.pone.0062681 (PMC3646847; doi:10.1371/journal.pone.0062681)
Supplement: Table S5 — Real-time PCR primer sequences for candidate miRNAs. (DOC) [file pone.0062681.s007.doc]

**Table S5. Real-time PCR primer sequences for candidate miRNAs**

| **miRNA** | **Primer** |
| --- | --- |
| miRNA166 | TCGGACCAGGCTTCATTCCCC |
| miRNA167 | TGAAGCTGCCAGCATGATCT |
| miRNA319 | TTGGACTGAAGGGAGCTCCC |
| miRNA399 | GCCAAAGGAGATTTGCCCCG |
| Pto-F11 | ACGAGCCATCATAACTGTAGG |
| Pto-F19 | CTCAGATTAGCCAGGTGCCT |
| Pto-F25 | TATAGATTGCAGAGGGAACC |
| Pto-F28 | TTGGGCTGGCAGTTGTGATGAC |
| Pto-F36 | TGGCCCATGATCTTCATTGTG |
| Pto-F14 | GCATTTGGACGTCGGGGAACT |
| Pto-F16 | TAATTCCATGACTGTGTACAG |
| Pto-F45 | TGTCCTGACTCGAACTCGAGA |
| Pto-F47 | GGGACTGCTGTAGATGCTTGG |
| Pto-F51 | TTGGAATCCTCTCTGATAATGC |
| Pto-F54 | TTGGAGCTCGAGACTTGGCAC |
| Pto-F55 | ATCACTGTGTCTATTAGGATGG |
| Pto-F56 | TGCTAGGACCAAGTTTTCTGG |
| Pto-F58a | TGAATGGCTGTTGAACTTGGC |
| Pto-F6 | TGTGGTAGATATGTGAGGATT |
| Pto-F61 | TTATAGTTTTGAAATCCGGCC |
| Pto-F64 | CAACACCAGACCCAAGAGCTT |
| Pto-F65 | TTAGGGTTTAGGGTTTAGAA |
| Pto-F66 | TCGAGATTGTACTGTTCATA |
| Pto-F68 | GTGGGTGGGTCTGGGTGGCA |
| Pto-F7 | CTCAAACTTTTCTCTTACTTC |
| Pto-F73 | TGTTTTCCGGAAAGTAGTTTC |
| Pto-F76 | TCGGCGTTGATGTAGAATGGC |
| Poly(T) adapter | GCGAGCACAGAATTAATACGACTCACTATAGG(T)12VN* |
| Reverse primer | GCGAGCACAGAATTAATACGAC |
| 5.8S rRNA | GTCTGCCTGGGTGTCACGCAA |

*V = A, G, C; N = A, T, G, C.
