# Supplementary material for: Sexual Dimorphism Floral MicroRNA Profiling and Target Gene Expression in Andromonoecious Poplar (Populus tomentosa)
Source: PLoS One. 2013 May 7;8(5):e62681. doi: 10.1371/journal.pone.0062681 (PMC3646847; doi:10.1371/journal.pone.0062681)
Supplement: Table S6 — Real-time PCR primer sequences for candidate target genes. (DOC) [file pone.0062681.s008.doc]

***Table S6. Candidate target genes real time PCR primer sequences***

| **Target Gene** | **Sequences (5'to3')** | **Tm(℃)** | **Size(bp)** | **Efficiency (%)** |
| --- | --- | --- | --- | --- |
| POPTR_0001s16100 | F: AGCTTGGTTGAGAGCATGGAA  R: GCACGTTGAGCTTGCTCTTCT | 58 | 67 | 99.2~104.2 |
| POPTR_0015s07740 | F: GGATAAAATCGACTGGCAAGCT  R: TGTGGCAAGTCGGCTACGA | 58 | 70 | 99.5~105.9 |
| POPTR_0008s10610 | F: CTGGGCCATTGAAACAGTATACC  R: GGTTTTGCTGCCATCTTCGA | 58 | 64 | 95.9~104.9 |
| POPTR_0013s00750 | F: ACGCGGGATGCAAAGTCA  R: GACCTCGTGAAGGAATCTCAGAA | 58 | 68 | 98.8~105.6 |
| POPTR_0007s12710 | F: GGAGGCATGCTCTCTACCCTTT  R: ACGTCTGCACCGCATCCT | 58 | 61 | 97.1~101.1 |
| POPTR_0004s10800 | F: CTTTTCTCCAGCACTGCTATCCA  R: CTTGCCTCCCTATCCATTGG | 58 | 62 | 98.8~104.3 |
| POPTR_0017s14410 | F: TGGAGTGGTGCCAGAATCAG  R: GGAGCACTTTGGTGCGAGAT | 58 | 57 | 99.8~102.3 |
| POPTR_0002s02970 | F: GGTAGGGCAAAGTGGGTTCA  R: CTGCCAACCTTCTGTTAGCTTTC | 58 | 68 | 97.5~105.4 |
| POPTR_0010s13490 | F: GCAGAGAAGAGGCCCAGGAT  R: TTTCCGGCGAAGGTTGTC | 58 | 59 | 95.9~104.9 |
| POPTR_0007s10780 | F: CTACGAGGCAGGTTGTTTTTCA  R: ATTTTCTTGGTGGTGGTTCTCTCT | 58 | 67 | 98.8~105.6 |
| POPTR_0005s18400 | F: CTCCCCAGTTTGCAAATGCT  R: AGCCAGTTTTGTGGAGTCCTAATC | 58 | 70 | 97.1~101.1 |
| POPTR_0010s11350 | F: GGGATGGATGGATACGTCTCA  R: CTGGCAACTGCCTGATAGAGATT | 58 | 62 | 98.8~104.3 |
| POPTR_0008s13720 | F: AAGAGTGGGATGGATGGATATGTC  R: TGGCAACTGCCTGATAGAGATTT | 58 | 67 | 99.8~102.3 |
| POPTR_0006s23760 | F: CGATCCCCTGGCCATTCT  R: GCTAGAGTTGGGAGATCCTCTTGA | 58 | 62 | 97.5~105.4 |
| POPTR_0007s08330 | F: GAGCAGTCAATATACATGCAAGCA  R: TGGATCAGTTCTCACACGGTTT | 58 | 89 | 97.5~100.7 |
| POPTR_0002s25330 | F: CAGGGCCCAAACCAGAGA  R: TTGCATGCGAGTTTCTTTGC | 58 | 57 | 98.6~101.2 |
| POPTR_0001s40770 | F: CCACTCGATCTCACCCTTAACAG  R: AACTAACCCCATCTGGGAACTG | 58 | 70 | 97.2~103.1 |
| POPTR_0018s06080 | F: CGCGGCTGGAGAAGAAGA  R: CTTTGGCTGCTCATTAATCAGCTT | 58 | 62 | 99.1~101.9 |
| POPTR_0013s01640 | F: TCTACGTCGGACGCATACGA  R: TTCGCCGTAAGGAGAAGTAGGA | 58 | 69 | 97.9~102.1 |
| POPTR_0012s04470 | F: GACAGAGAAAAGCAGCCATGGT  R: TTACAACTTCTCGAGGCAAAAGAA | 58 | 67 | 98.2~105.2 |
| POPTR_0005s05550 | F: ATCGAGGTGCACTACTCTGAGCTA  R: TGCAACAGGTCCATGATTGATAC | 58 | 68 | 99.3~100.9 |
| POPTR_0004s17150 | F: CAAACAGCAGTCCTCAAGCAAA  R: TCATAGCCATGTTTCTTGCCTAAA | 58 | 65 | 99.9~106.8 |
| POPTR_0008s10100 | F: GCTGCTTATGCTATGCGACTTG  R: CTCGTCTCCTTCCATCCTGAAT | 58 | 63 | 98.8~103.2 |
| POPTR_0005s14640 | F: CTGCTCAGCAACATCACACAAG  R: TCGCACAGCATTTCAGACAGA | 58 | 59 | 97.8~104.3 |
| POPTR_0006s04590 | F: CTGTCCTTCCTTTCCTCATTGC  R: TGGACGACGGCCGTTTT | 58 | 60 | 99.7~108.1 |
| POPTR_0010s13870 | F: CATCCTACCAGGCTCACCTCTT  R: TTCCAATCAACTGGGATATGCA | 58 | 69 | 97.7~102.5 |
| POPTR_0002s02680 | F: GGCAGAGGTGAACAAAATGAAAG  R: TCTAGGTTTCCCATGGCAGGTA | 58 | 70 | 98.9~105.4 |
| POPTR_0004s18020 | F: GGTGAATGTTTTCCACTCATCGA  R: ACCGAGGAAAGAAACGAAATCA | 58 | 71 | 98.8~104.8 |
| POPTR_0019s15030 | F: GCCCTGGTCCAAGCATCA  R: CTGCAGAAGTGCTCGTTTGC | 58 | 59 | 97.7~103.4 |
| POPTR_0012s03530 | F: GAAATGCAGGGAGACAATGCA  R: AAAGGGTCCACCAAGGTATGG | 58 | 64 | 98.9~102.1 |

**Efficiency (%) represents the** [**amplification**](app:ds:amplification) **efficiency of each primer in qRT-PCR.**
